# Attitude and awareness of dental patients towards disclosure of medical history to dentists: a cross-sectional study

**DOI:** 10.3389/froh.2026.1748465

**Published:** 2026-04-15

**Authors:** Shahinaz N. Sembawa, Eman Dustakir, Bayan Skatawi, Nadin Alsulayhabi, Majd Alghamdi

**Affiliations:** 1 Division of Dental Public Health, Department of Preventive Dentistry, College of Dental Medicine, Umm Al-Qura University, Makkah, Saudi Arabia; 2 Faculty of Dentistry, Umm Al-Qura University, Makkah, Saudi Arabia

**Keywords:** attitude, awareness, dentist, disclosure, medical history

## Abstract

**Background:**

Patients’ willingness to share their medical history is vital, yet some may withhold information due to various reasons.

**Aim:**

To explore the attitude and awareness of dental patients towards the disclosure of medical history to their dentists in Makkah, Saudi Arabia.

**Methodology:**

A cross-sectional survey developed using Google Form was utilized for data collection. Adult Arabic or English-speaking patients who agreed to participate in the study were recruited. The questionnaire consisted of three parts: demographic information, patients’ attitude towards medical history disclosure (yes/no questions), and patients’ awareness about the importance of medical history (Likert-type scale). Data was analyzed using SPSS version 29. Descriptive statistics, Chi-square test, Fisher's Exact test, Kruskal Wallis and Mann–Whitney *U*-Tests were used for data analysis.

**Result:**

A total of 622 responses were received**.** Most participants were females (67.5%), hold bachelor's degrees (66.9%), and aged 18–24(29.4%). The majority of participants (94.2%) had prior dental treatment and (97.1%) did not experience clinical complications. Moreover, (76.4%) of the participants reported it's very important to inform the dentist about complications previously experienced in the dental clinic. However, few participants think it is very important to choose morning appointments for diabetic patients (33.9%). Also, the respondents reported that it is very important to tell the dentist about the month of pregnancy (71.2%).

**Conclusion:**

The findings suggest that adult dental patients in the current sample demonstrate a positive attitude towards disclosure of medical history to dentists. However, gaps in health awareness were identified. These findings highlight the potential role of patient education and interprofessional collaboration in improving patients’ health literacy.

## 1 Introduction

Patients' disclosure of their medical history is the first step to proper management and diagnosis of dental patients. The disclosure of medical conditions, medications, allergies, and previous treatments is essential for dentists to make informed decisions, ensure patient safety, and prevent complications during dental procedures. However, significant discrepancies have been found regarding what is disclosed by patients (1). Despite its importance, patients may withhold or fail to disclose relevant medical information due to various factors such as lack of awareness, perceived irrelevance, embarrassment, or concerns about privacy (2).

A research investigation was conducted to ascertain the frequency of self-reported medical issues within a group of dental school patients at the Institute of Dental Sciences, situated in India. Comprehensive medical backgrounds were gathered from 3,786 new dental patients during their outpatient visits. Among the entire patient population, 38% disclosed a positive medical history related to at least one systemic health concern (3). This highlights the substantial burden of medical comorbidities in dental settings. However, multiple studies have demonstrated inconsistencies in what patients choose to report (1, 2).

Several studies reported positive patient attitudes and willingness for chairside medical screening. According to a study done in Riyadh, 61.3% of respondents reported their willingness to discuss their medical history with the dentist, and 76.1% said they would discuss any previous complications of their disease. Also, two thirds of the sample (71.9%) will provide information about drugs, and 76.1% will disclose the month of pregnancy (4). Another study reported that more than 80% of the patients always informed their dentist about their prescription medications, but this number fell to 71% for over-the-counter drugs (OTC) and even lower (to 63%) for herbal and dietary supplements (HDS). This shows that patients would tell their dentists about their medications depending on the type of medication If it is prescription medications, OTC or HDS (5). Moreover, this pattern suggests that patients may not perceive all categories of medical information as equally relevant to dental care.

Another study reported that only one in three respondents reported telling their conventional healthcare practitioner they used (HDS). They discovered that only 39% of people who use prescription drugs and only 44% of adults with two or more chronic medical conditions disclosed to their healthcare provider that they use (HDS) (6).

The importance of accurate medical history disclosure in ensuring patients' safety and effective dental care is widely recognised. However, patients' understanding of its significance and their willingness to disclose relevant information remain variable. In the Saudi Arabian context, factors such as differences in health literacy, the coexistence of public and private dental care systems, and cultural influences on patient–dentist communication may affect disclosure attitudes. Additionally, the diverse and dynamic population of the city of Makkah further necessitates a better understanding of patient perspectives in this setting. Limited evidence is available in Saudi Arabia regarding dental patients' attitude towards medical history disclosure and their awareness of the importance of sharing such information with dentists.

Therefore, this study aims to assess the attitudes and awareness of adult dental patients in Makkah, Saudi Arabia, regarding the disclosure of medical history to dentists, and to evaluate factors associated with these attitudes in order to inform patient safety and dental care practices.

## 2 Material and methods

A cross-sectional study design was employed utilizing an online questionnaire to explore the awareness and attitude of patients towards disclosure of medical history to the dentist. This study has been approved by The Biomedical Research Ethics Committee at Umm Al-Qura University with the approval number (HAPO-02-K-012-2023-11-1899) dated 27th of November, 2023.

### 2.1 Participants' recruitment

A convenience sample of patients was recruited from the dental teaching hospital at Umm Al-Qura University. Additional recruitment took place by the research team among individuals meeting the inclusion criteria outside the hospital settings. Inclusion criteria were adult dental patients or individuals with previous dental treatment experience, speaking Arabic or English and consenting to participate in the study. Patients who don't speak English or Arabic, patients under 18 years old, and who don't accept to participate in the study were excluded.

### 2.2 The questionnaire

The questionnaire was adapted from a previously validated instrument (4) and translated into Arabic to suit the study population. Reverse translation was performed, and the translated version was reviewed to ensure accuracy and linguistic equivalence prior to data collection. The final version was developed and administered electronically using Google Forms.

The questionnaire is composed of three major sections. The first section contained demographic data, including age, gender, marital status, educational level, and occupation. The second section assessed the participants' previous disclosure-related experience and awareness using “yes or no” closed-ended questions. The third section assessed patient awareness of the importance of disclosing medical history to their treating dentists. It employed a five-point Likert-type scale indicating measures of very important, important, neutral, unimportant, and totally unimportant.

### 2.3 Data analysis

The sample size was calculated using Epitools online calculator according to 80% power, 95% confidence interval and 5% margin of error. Therefore, the minimum required sample size to achieve statistical precision is 323 (7). However, recruitment yielded 622 eligible participants; all were included to increase statistical precision.

Data was collected, summarized, and presented in tables using Excel, and statistical analysis was performed using SPSS (version 29) (8). Descriptive statistics including frequency, percentage, mean and median were used to describe the data. For comparisons involving categorical (yes/no) responses, the Chi-square test was used, and Fisher's exact test was applied when expected cell counts were small. Associations between Likert-scale responses and demographic variables were assessed using non-parametric tests due to the ordinal nature of the data. The Mann–Whitney *U*-test was used for comparisons between two groups (gender), while the Kruskal–Wallis test was applied when comparing more than two groups (age categories, education level and employment sector).

## 3 Results

A total of 622 participants completed the survey. Most participants were females (67.5%), aged 18–24 (29.4%), married (54.7%), hold bachelor's degrees (66.9%) and unemployed (49.7%). The sociodemographic characteristics of the sample are summarized in (Table 1).

**Table 1:** Sociodemographic characteristics of the sample(*n* = 622).

Variable	Frequency (percent)
Gender	
Male	202 (32.5%)
Female	420 (67.5%)
Age	
18–24	183 (29.4%)
25–34	120 (19.3%)
35–44	97 (15.6%)
45–54	109 (17.2%)
55 and older	113 (18.2%)
Marital status	
Single	241 (38.7%)
Married	340 (54.7%)
Widow	13 (2.1%)
Divorced	28 (4.5%)
Education	
High school	125 (20.1%)
Bachelor’s degree	416 (66.9%)
Postgraduate degree	43 (6.9%)
Other	38 (6.1%)
Sector of work	
Unemployed	309 (49.7%)
Governmental	108 (17.4%)
Private	52 (8.4%)
Healthcare	16 (2.6%)
Other	137 (13.8%)

Almost all participants (94.2%) had previously received dental treatment, which was significant with occupation (*p* = 0.013). Most of the participants did not have any complications in the clinic due to non-disclosure of high blood pressure (97.1%), which was significant with age (*p* = 0.039). However, the majority of the participants believe it is not safe to treat teeth during pregnancy (63.3%). Moreover, most of the participants did not receive instructions about the appropriate period to receive dental treatment during pregnancy (78.1%) and this was significant with age (*p* < 0.001), gender (*p* < 0.001), marital status (*p* < 0.001) and occupation (*p* < 0.001) (Table 2).

**Table 2:** Participants’ previous disclosure-related experience and awareness (*n* = 622).

Statement	Yes [n (%)]	No [n (%)]
Have you received dental treatment before?	586 (94.2%)	36 (5.8%)
Has a doctor ever told you that some oral side effects may appear from any disease or medication such as ulcers or bleeding?	201 (32.3%)	421 (67.7%)
Have you ever had complications in the clinic because you did not tell the doctor about your high blood pressure?	18 (2.9%)	604 (97.1%)
Have you ever been alerted to any of the medicines/analgesics that are contraindicated, or should the dose be adjusted during dental treatment?	166 (26.7%)	456 (73.3%)
Have you been given any instructions regarding the appropriate period to treat teeth during pregnancy?	136 (21.9%)	486 (78.1%)
Do you think it is safe to treat teeth during pregnancy?	228 (36.7%)	394 (63.3%)

Most of the participants reported that they were never told about some oral side effects (ulcers and bleeding) that may appear from disease or medication (67.7%). Moreover, (73.3%) of the participants were not aware about medicines or analgesics that are contraindicated or need dosage adjustment during dental treatment, which was significant with gender (*p* = 0.004), age and marital status (*p* = 0.015) (Table 2).

Majority of the participants think it's very important to inform the dentist about complications previously experienced in the dental clinic such as fainting/losing consciousness (76.4%) (Median =1, IQR:1), and it was significant with gender (*p* = 0.002), age (*p* = 0.048), marital status (*p* = 0.043) and education (*p* = 0.009). Also, the respondents reported that it's very important to tell the dentist about the month of pregnancy (71.2%) (Median=1, IQR:1-3), and it was significant with gender (*p* = 0.006), occupation(*p* = 0.006), age (*p* = 0.001) and marital status(*p* = 0.001). Additionally, (71.2%) think it's very important to inform the dentist about any disease before performing dental treatments (Median=1, IQR:1-2) and it was significant with age(*p* = 0.001), marital status(*p* = 0.001) and occupation(*p* = 0.009). Moreover, some patients think it is very important to inform the dentist if they have any allergies before dental treatment (67.8%)(Median=1, IQR:1-2).Also, the respondents think it is very important to inform their dentist about their HbA1c levels for the diabetic patients (67.2%)(Median=1, IQR:1-2) which was significant with age(*p* = 0.001), marital status (*p* = 0.001) and occupation(*p* = 0.001).

On the other hand, few participants reported it is very important to choose morning appointments for diabetic patients (33.9%) (Median = 2, IQR:1-3) and it was significant with age(*p* = 0.001), marital status (*p* = 0.007) and occupation(*p* = 0.001). Also, few participants responded it's important to ask diabetic patients to bring a piece of sugar to the dental clinic (36.3%) (Median=2, IQR:1-3) and it was significant with age (*p* = 0.049) (Table 3).

**Table 3:** Participants’ awareness of the importance of medical history disclosure (*n* = 622).

Statement	Very important	Important	Not sure	Unimportant	Totally unimportant
How important it is to inform the dentist about complications previously experienced in the dental clinic such as fainting/losing consciousness?	475 (76.4%)	99 (15.9%)	35 (5.6%)	6 (1%)	7 (1.1%)
How important is telling the dentist about the month of pregnancy?	443 (71.2%)	89 (14.3%)	70 (11.3%)	10 (1.6%)	10 (1.6%)
How important is it to bring indispensable medicines like an asthma inhaler for a dental appointment?	307 (49.4%)	144 (23.2%)	132 (21.2%)	29 (4.7%)	10 (1.5%)
Do you think telling your dentist about any disease before performing dental treatments is?	443 (71.2%)	137 (22.1%)	43 (6.9%)	15 (2.4%)	4 (0.8%)
How important is it to complete the list of all names and dosages of the medicines you are taking?	290 (46.6%)	180 (28.9%)	108 (17.4%)	36 (5.8%)	8 (1.3%)
Do you think giving the dentist the name and number of the doctor supervising your medical condition is?	194 (31.2%)	183 (29.4%)	151 (24.3%)	81 (13%)	13 (2.1%)
How important is it for the dentist to monitor the medical conditions of the patient?	308 (49.5%)	228 (36.7%)	59 (9%)	25 (4%)	5 (0.8%)
How important is it to inform the dentist if you have any allergies before treatment?	422 (67.8%)	143 (23%)	42 (6.8%)	13 (2.1%)	2 (0.3%)
How important is it for diabetics to inform their dentist about their HbA1c levels?	418 (67.2%)	115 (18.5%)	67 (10.8%)	16 (2.5%)	6 (1%)
How important is it for a dentist to examine health conditions if they suspect a disease?	357 (57.4%)	178 (28.6%)	64 (10.3%)	17 (2.7)	6 (1%)
How important is it to speak with a doctor, such as a cardiologist, about any dental treatment?	297 (47.7%)	141 (22.7%)	137 (22%)	33 (5.3%)	14 (2.3%)
How important is it to ask the dentist about the availability of oxygen and nitroglycerin for heart patients?	265 (42.6%)	146 (23.5%)	185 (29.7%)	19 (3.1%)	7 (1.1%)
How important is it for diabetics to choose morning appointments?	211 (33.9%)	128 (20.6%)	248 (39.9%)	28 (4.5%)	7 (1.1%)
How important is choosing the right time for dental treatment after a heart attack?	287 (46.2%)	99 (15.9%)	221 (35.5%)	6 (1%)	9 (1.4%)
How important is asking diabetics to bring a piece of sugar to the dental clinic?	226(36.3%)	143(23%)	215(34.6%)	42(3.9%)	14(2.2%)

## 4 Discussion

This study aimed to explore the attitude and awareness of adult dental patients in Makkah, Saudi Arabia regarding the disclosure of medical history to dentists. The majority of the participants showed a positive attitude towards disclosure of medical history to their dentists. However, gaps in health awareness were identified, which might interfere with the participants' ability to disclose relevant medical information. The findings suggest that disclosure behavior may vary according to demographic factors such as gender, age, educational level and occupation.

Findings of the current study revealed that most of the participants have a positive attitude towards disclosing their medical history to their dentists. This conforms with the results of an earlier study conducted in the USA, where almost 90% of patients responded that it is important for dentists to screen for medical conditions (9). On the other hand, a study from Malaysia reported that almost half of their sample of patients living with HIV were reluctant to disclose their HIV status. This reluctance was due to several factors like personal rights and concerns about inconveniences, including treatment refusal and discrimination (10). Another study from the US reported failure of patients facing eminent threats to disclose their information to clinicians due to embarrassment, not wanting the information in their medical record, not wanting to have to engage in a difficult follow-up behavior and not wanting to be judged or lectured (11).

The majority of the participants in the current study reported the importance of telling the dentist about any disease before performing dental treatments and the importance of examining health conditions by the dentist if they suspect a disease**.** A Study demonstrated that majority of the respondents in primary care clinics and general practices are willing to be screened for high blood pressure (83% vs. 74%) and diabetes (82% vs. 72%) and are willing to share the results with the dentist (12). While another study in the U.S reported that 49% of hypertensive patients in the sample were not aware of their high blood pressure prior to their dental visit. Therefore, screening dental patients routinely for hypertension and DM is necessary (13).

On the other hand, the participants were not sure if hypertensive patients need to carefully choose the right time for dental treatment after a heart attack, or if a diabetic patient should choose morning appointments and bring a piece of sugar to the appointment. However, 47.7% of patients believe that it is very important to talk to a cardiologist about any dental treatment. Therefore, the dentist must educate patients with chronic diseases such as diabetes and hypertension about the well-known complications that could arise during dental treatment (4). Moreover, effective collaboration and communication between dentists and physicians for careful planning regarding the time and nature of dental procedures is essential to ensure the safety of such patients.

Although most participants agreed that informing the dentist about pregnancy is important, our study revealed that only 36.7% agree that it is safe to treat teeth during pregnancy. Moreover, only 21.9% reported receiving instructions about the appropriate period to treat teeth during pregnancy. Similar findings have been reported in a study done in Riyadh city (4). Many pregnant women were found to hold negative beliefs about dental care, regardless of the treatment type. Understanding these beliefs and their contributing factors are essential for developing preventive interventions and promoting oral health awareness during pregnancy. These negative perceptions may arise from a lack of information and awareness among pregnant women and their primary care providers (14). This suggests a need for patient education and updates from both obstetricians and dentists.

The majority of the participants reported the importance of telling the dentists about having any allergies before treatment. Saleem et al. (15) revealed that 59.7% of dental students reported asking patients about latex allergies before rubber dam use, showing awareness of protocol. Patients' sensitivity to latex was the main reason for not using rubber dam. Proper precautions, like using non-powdered gloves and good ventilation, are essential to avoid latex contact with allergic patients. Though uncommon, latex allergies require screening questions about atopy, including hay fever, eczema, and food allergies (16). Moreover, most of the participants in this study (75%) are aware of the importance of disclosing the names and dosages of the medicines they are taking to their dentists. This conforms with the findings of an American study, where most of the responding patients reported always informing their dentists of their prescribed, OTC, and herbal/supplement medications and their understanding of its importance (5).

Our investigation is in agreement with previous literature that demographic factors such as age, education, occupation, and marital status are key factors influencing patients disclosure of their medical history to their dentists (4). Disparities in disclosure might be explained by differences in the needs, barriers, and preferences for health education. The importance of transparency should be emphasized through health education to ensure patient safety (17).

The large sample size yielded by the data collection is considered a strength of the current study. Additionally, a validated questionnaire was used, available in both Arabic and English languages, enhancing the accuracy and validity of our findings.

However, it's important to acknowledge the limitations of this study. First, findings of the study may not be fully generalisable due to the cross-sectional design and predominance of females and relatively highly educated participants. Disclosure attitude may vary by gender, while higher education may lead to over estimation of the participants' positive attitude and health awareness. Second, using a convenience sampling approach may introduce selection bias, as health awareness and engagement of participants who chose to respond may differ systematically from those who did not. Moreover, this sampling approach precluded calculating the response rate due to the absence of a defined sampling frame. Third, data were collected through an online survey, which may lead to response bias, as individuals with greater access to digital platforms or higher digital literacy are more likely to participate. Furthermore, the reliance on patients' self-reports introduces the potential for recall and social desirability biases. Lastly, given that the validation methods of the original instrument were not reported and the internal consistency of the adapted version was not assessed, the reliability of the instrument in the current population cannot be fully established. Future research with a more representative sample could employ multivariable analysis to control for potential confounding factors that may influence patients' disclosure attitudes.

## 5 Conclusion

The findings suggest that adult dental patients in the current sample demonstrate a positive attitude towards disclosure of medical history to dentists. However, gaps in health awareness were identified. These findings highlight the potential role of patient education and interprofessional collaboration in improving patients' health literacy. Nevertheless, the findings should be interpreted within the context of the current study, given the cross-sectional design and the predominance of females and highly educated participants that may affect disclosure behavior and generalizability of the results.

## Data Availability

The original contributions presented in the study are included in the article/Supplementary Material, further inquiries can be directed to the corresponding author.
